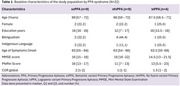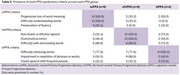# Assessing the Overlap in Diagnostic Criteria for Primary Progressive Aphasia in a Latin American Population

**DOI:** 10.1002/alz70857_104590

**Published:** 2025-12-25

**Authors:** Diego Bustamante‐Paytan, José Carlos Huilca, Gregory Brown, Maria Fe Albujar‐Pereira, Katherine Aguero, Graciet Verastegui, Zadith Yauri, Pamela Bartolo, Daniela Bendezu, Karol Melissa Lipa‐Pari, Rosa Montesinos, Nilton Custodio

**Affiliations:** ^1^ Unidad de Investigación de Deterioro Cognitivo y Prevención de Demencia, Instituto Peruano de Neurociencias, Lima, Lima, Peru; ^2^ Unidad de Investigación de Deterioro Cognitivo y Prevención de Demencia, Instituto Peruano de Neurociencias, Lima, Peru, Lima, Lima, Peru; ^3^ University of California, San Francisco, San Francisco, CA, USA; ^4^ Instituto Peruano de Neurociencias, Lima, Lima, Peru; ^5^ Unidad de Investigación de Deterioro Cognitivo y Prevención de Demencia, Instituto Peruano de Neurociencias, Lima, Peru; ^6^ Equilibria, Lima, Lima, Peru; ^7^ Hospital Nacional Cayetano Heredia, Lima, Lima, Peru; ^8^ Unidad de Investigación y Docencia, Equilibria, Lima, Peru., Lima, Lima, Peru; ^9^ Unidad de Investigación y Docencia, Equilibria, Lima, Lima, Peru

## Abstract

**Background:**

Primary progressive aphasias (PPA) are characterized by a progressive deterioration of language. Despite the establishment of international criteria in 2011 defining three variants (semantic, nonfluent, and logopenic), these variants can present overlapping symptoms, making their identification and differentiation challenging. This study analyzes the presence of specific symptoms of each variant among patients diagnosed with different PPA syndromes.

**Method:**

A retrospective study was conducted on patients diagnosed with PPA recruited between Jun 2021 and November 2024 at the Department of Neurology of the Instituto Peruano de Neurociencias, Lima, Peru. The inclusion criteria for the clinical group were as follows: (1) PPA diagnosed and classified according to Gorno‐Tempini et al. (2011), (2) Spanish‐speaking individuals, (3) aged between 50 and 80 years. The diagnostic gold standard was determined by applying the diagnostic criteria of Gorno‐Tempini et al. (2011) based on the results of a comprehensive neuropsychological assessment battery, as well as the findings from MRI. The exclusion criteria included: (1) severe language impairment that hinders the ability to follow instructions, (2) uncompensated auditory or visual impairments, (3) previous neurological history involving acquired lesions or neurodevelopmental disorders. Descriptive statistics were used to compare the distribution of characteristic symptoms of each variant within the diagnostic groups.

**Result:**

Twenty‐two patients were included in the analysis. The median age was 69 years for the semantic variant (svPPA), 66 years for the nonfluent variant (nfvPPA), and 67.5 years for the logopenic variant (lvPPA). Nearly half of the patients in the nfvPPA group were bilingual, while a low proportion of indigenous language speakers (Quechua or Aymara) was observed across all three groups. Only 55% of svPPA patients exhibited preserved fluency. A high proportion of patients with svPPA and nfvPPA had difficulties retrieving words (77.8% in each case). Furthermore, more than half of the lvPPA patients exhibited symptoms consistent with nfvPPA criteria.

**Conclusion:**

This study observed a high frequency of symptom overlaps among the PPA variants. Future studies are needed to establish more accurate diagnostic criteria, particularly in contexts with cultural and linguistic differences like Latin America.